# Anti-idiotypic antibodies: a new approach in prion research

**DOI:** 10.1186/1471-2172-10-16

**Published:** 2009-03-19

**Authors:** Anja Colja Venturini, Maja Bresjanac, Tanja Vranac, Simon Koren, Mojca Narat, Mara Popović, Vladka Čurin Šerbec

**Affiliations:** 1 Department for Production of Diagnostic Reagents and Research, Blood Transfusion Centre of Slovenia, Ljubljana, Slovenia; 2 Institute of Pathophisiology, Medical Faculty, University of Ljubljana, Ljubljana, Slovenia; 3 Faculty of Chemistry and Chemical Technology, University of Ljubljana, Ljubljana, Slovenia; 4 Department of Animal Science, Biotechnical Faculty, University of Ljubljana, Domžale, Slovenia; 5 Institute of Pathology, Medical Faculty, University of Ljubljana, Ljubljana, Slovenia

## Abstract

**Background:**

In certain cases, anti-idiotypic antibodies that recognize an antigen-combining site of an antibody can mimic the structure and/or function of certain nominal antigens. This feature makes them particularly useful if conventional experimental approaches fail to fulfil expectations, especially when the molecule of interest is infectious, toxic or difficult to isolate and purify. We suggest the application of an anti-idiotype concept to the field of prion biology, with the aim of evoking a humoral immune response against the pathological isoform of the prion protein (PrP^Sc^). Different ways to induce anti-idiotypic responses were studied in mice and chickens using various forms of V5B2, a PrP^Sc^-specific monoclonal antibody we have described previously.

**Results:**

The preparation of anti-idiotypic monoclonal antibodies was achieved with well-defined strategies of immunization, selection and subsequent characterization. Our results demonstrate that it is possible to induce a strong anti-idiotypic immune response against the V5B2 monoclonal antibody in both xenogeneic and syngeneic experimental systems. From the competition seen between polyclonal and monoclonal anti-idiotypic antibodies and the original immunogen, the P1 peptide, and even more importantly, the ultimate target antigen, PrP^Sc^, we conclude that selected antibodies bind to the antigen-combining site of the V5B2 monoclonal antibody and might even resemble the PrP^Sc^-specific epitope. The involvement of both antigen-combining sites in the interaction between V5B2 and the most promising monoclonal anti-idiotypic antibody was further supported by molecular docking.

**Conclusion:**

The results of the present study not only provide an example of the successful production of Ab2 monoclonal antibodies based on a well planned strategy for selection, but should also provide a new experimental approach that is applicable to the field of prion diseases.

## Background

According to the Network Theory of Niels Jerne, the immune system is a network of interacting idiotypes that is involved in the regulation of immune responses [1]. Anti-idiotypic (Ab2) antibodies are a special set of antibodies that can react with idiotopes, which represent unique antigenic determinants on the surface of an antibody. Each antibody constitutes a small set of idiotopes that form its own idiotype. Private idiotopes have been shown to be associated with the complementarity-determining regions (CDRs), which, in addition to various rearrangements of V-(D)-J gene segments, also reflect random somatic mutations and/or N-region additions with a low probability of repetition in another individual. Unlike private idiotopes, recurrent idiotopes are encoded by germline genes, which can generally tolerate some somatic mutations without the loss of the original idiotope [2]. A single idiotope can stretch over a part of the CDR and a part of the framework region, as well as over both the light and heavy chain residues.

Ab2 antibodies can be classified into three distinct groups: the Ab2α antibody group are conventional antibodies that recognize idiotopes distinct from the antigen-combining site on primary Ab1 antibodies; Ab2β antibodies are internal image antibodies that recognize epitopes within the antigen-combining site and that resemble the nominal antigen (internal image); and Ab2γ antibodies recognize epitopes within the antigen-combining site, but do not resemble the nominal antigen [3].

The most intriguing group of Ab2 antibodies are those of Ab2β, the internal image antibodies, which are directed against the binding site of the eliciting antibodies and can, in their paratope, structurally and/or functionally mimic the original antigen, or more precisely, the epitope of the original antigen [4-8]. This feature has led to the idea of using internal image antibodies as surrogate antigens for the development of active vaccines. Such an approach is especially useful when the hypothetically protective antigens are infectious, toxic or difficult to isolate and purify, as is the case in prion disease vaccine development.

Prion diseases, which are also known as transmissible spongiform encephalopathies (TSEs), are a group of incurable, fatal neurodegenerative diseases that affect humans and animals [9,10]. According to the widely accepted "protein only" hypothesis proposed by Stanley B. Prusiner, TSEs are caused by misfolding of the normal cellular prion protein (PrP^C^) into the protease-resistant isoform, PrP^Sc^, which then accumulates in the central nervous system [10,11]. Since the epidemic of bovine spongiform encephalopathy (BSE) in the nineties and the transmission of the disease to humans as variant Creutzfeldt-Jakob disease (vCJD) [12,13], significant scientific resources have been devoted to improve our understanding of prion biology. Interestingly, to date, it has not been possible to detect a significant anti-PrP^Sc ^immune response during the course of prion diseases [10,14,15]. Similar difficulties emerge with attempts to experimentally evoke a protective anti-PrP^Sc ^immune response in wild-type animals, when the recombinant prion protein or peptides derived from the amino-acid structure of the prion protein are used as antigens for immunization [16-21]. Since the prion protein is a highly conserved ubiquitous protein, it induces strong B-cell and T-cell immune tolerance when introduced into an organism [20,22].

As an alternative to conventional active anti-prion vaccine development, the Ab2 approach offers another way to overcome the unresponsiveness of the immune system. For the development of Ab2 antibodies that mimic the PrP^Sc^-specific epitope, a PrP^Sc^-specific antibody is a prerequisite. In our previous studies, we described and characterized V5B2, a PrP^Sc^-specific monoclonal antibody, and indicated its potential applicability for diagnostic purposes [23,24]. Based on biophysical studies, it has been proposed that V5B2 recognizes an epitope on PrP^Sc ^that is similar or identical to the conformation of the P1 peptide in solution (predominantly a dimeric/oligomeric form) and in fibril-like aggregates, both of which differ from the conformation of the corresponding region in PrP^C ^[25].

According to the internal image phenomena, Ab2β antibodies against the V5B2 paratope would carry the PrP^Sc^-specific epitope image in their antigen-combining site, thereby providing an insight into its structure and maybe even serving as a tool for idiotypic vaccine studies.

The aim of this study was thus to investigate the applicable ways to induce an anti-idiotypic response to the PrP^Sc^-specific monoclonal antibody in xenogeneic and syngeneic experimental systems, and to define the preparation of Ab2 monoclonal antibodies with well-defined strategies for immunization, selection and subsequent characterization.

## Results

To evoke an immune response against the idiotype of the mouse V5B2 monoclonal antibody, two different models of experimental immunization were set up: the xenogeneic and syngeneic experimental systems.

### Anti-idiotypic response in chickens

**Figure 1:**
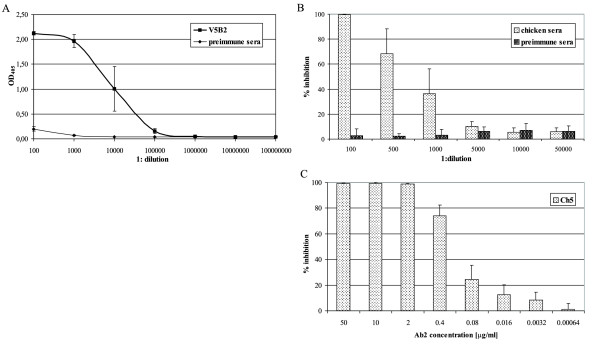
**The chicken immune response as monitored by detection of antigen-specific IgY**. (A) ELISA: chicken sera were tested at 10-fold dilution steps starting from a 1:100 dilution. The serum before immunization was used as the negative control. Each line represents the mean absorbance of 5 individual chicken sera ± standard deviation. (B) Competition assay for polyclonal chicken sera: for the competition assay, V5B2 was pre-incubated with the chicken sera that had been previously immunized with V5B2, and the subsequent binding ability of V5B2 to the P1 peptide was measured by ELISA. Each column represents the mean inhibition of V5B2 binding to the P1 peptide from the 4 individual chicken sera with the highest antibody titres ± standard deviation. (C) Competition assay for immunity purified chicken IgY (Ch5): each column represents the mean inhibition of V5B2 binding to the P1 peptide of at least three experiments ± standard deviation.

In the xenogeneic experimental model, the chickens were immunized with the mouse V5B2 monoclonal antibody. The humoral immune response was monitored by detection of antigen specific IgY in the immune sera after the 3^rd ^immunization by ELISA (Fig. 1A). In particular, the OD_405 _values were 2.10 ± 0.04, 1.97 ± 0.13 and 1.00 ± 0.45 at the 100-, 1,000- and 10,000-fold dilutions, respectively. Hardly any signal could be detected at dilutions ≥ 100,000-fold (OD_405 _0.15 ± 0.05). Since our specific interest was restricted to Ab2 antibodies only, the competition between chicken Ab2 antibodies and the original antigen, the P1 peptide, for binding to the paratope of the V5B2 monoclonal antibody offered an insight into the Ab2 humoral response (Fig. 1B). The two chickens with sera that exhibited the highest inhibition were further immunized, although this time with the Fab fragment of V5B2, to induce proliferation of Fab V5B2-specific B-cell clones only. Unfortunately, none of the resulting hybridoma cell clones maintained specific IgY production (data not shown). To better characterize the potent polyclonal immune response against the idiotype region, the Fab V5B2 specific antibodies were purified from the chicken blood sera. As shown in Fig 1C, the Ch5 immunoaffinity-purified polyclonal Ab2 antibodies had an inhibitory capacity of 100% when used at the concentration of only 2 μg/ml, whereas it declined to around 10% at the concentration of 3.2 ng/ml.

### Anti-idiotypic response in syngeneic mice

**Figure 2:**
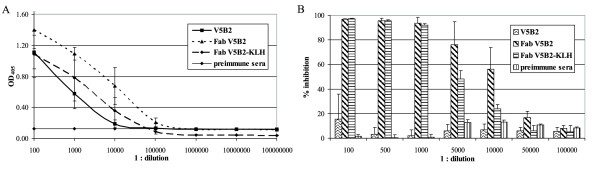
**The mice immune responses elicited against different antigens**. (A) ELISA: immune sera were tested at 10-fold dilution steps starting from a 1:100 dilution. Pre-immune serum was used as the negative control. Each line represents the mean absorbance of 5 individual mice sera ± standard deviation. The antigens used for the immunizations are listed in the upper right corner. (B) Competition assay: V5B2 was pre-incubated with the serially diluted sera of mice immunized with different forms of V5B2 (listed in the upper right corner) and the V5B2 binding to the P1 peptide was measured by ELISA. Each column represents the mean absorbance of 4 individual mice sera with the highest antibody titres ± standard deviation.

In the second experimental model, syngeneic BALB/c mice were challenged with three different forms of the same IgG1 molecule: the whole V5B2 monoclonal antibody, the Fab fragment of V5B2, and the Fab fragment of V5B2 covalently coupled to the highly immunogenic carrier molecule KLH. Ten days after the 3^rd ^immunization, blood was taken from the tail vein and the sera were tested for specific antibodies by indirect ELISA, and for Ab2 antibodies by competitive ELISA, as shown in Fig 2. The mean OD_405 _values at a 1:100 dilution were 1.11 ± 0.22, 1.40 ± 0.24 and 1.08 ± 0.3 for sera of mice immunized with V5B2, Fab V5B2 and Fab V5B2-KLH, respectively. The anti-V5B2 sera values declined faster with further dilutions, as compared to the anti-Fab V5B2 and anti-Fab V5B2-KLH sera, for which the OD_405_values were still 0.68 ± 0.24 and 0.35 ± 0.17, respectively, at a dilution 1:10,000. In agreement with these data, the most potent binding inhibition of V5B2 to the P1 peptide in ELISA was seen with the anti-Fab V5B2 and anti-Fab V5B2-KLH sera. More than 90% inhibition was reached with the anti-Fab V5B2 and anti-Fab V5B2-KLH sera diluted to 1:1,000, and more than 50% inhibition with these sera diluted 1:10,000 and 1:5,000, respectively. Since the highest immune response against Fab V5B2 and the strongest competition of Ab2 antibodies in immune sera were seen in mice immunized with Fab V5B2, the two mice with the highest anti-Fab V5B2 antibody titres were chosen and sacrificed for the cell fusion. In addition, the mouse with the highest anti-Fab V5B2 titre from the group of mice immunized with Fab V5B2-KLH was also selected for the cell fusion.

### Ab2 monoclonal antibody selection

The selection of murine Ab2 monoclonal antibodies was designed as a two-step process, including positive and negative selection. In the positive selection step, only hybridoma lines that reacted with Fab V5B2 in ELISA were selected for further characterization, whereas in the following negative selection step, only cell lines that did not bind to any of the negative selection proteins (the monoclonal antibodies and Fab fragments listed in Table 1) were chosen. In particular, the first protein used for negative selection was the Fab fragment from the E12/2 anti-recombinant bovine prion protein monoclonal antibody [26], which has a different epitope specificity but is of the same isotype as V5B2. This served to eliminate hypothetical isotype-specific antibodies that recognize the CH_1 _domain, the first constant domain, in addition to the variable domain that was also present in the Fab fragment. All of the further proteins used for negative selection were Fab fragments of monoclonal antibodies directed against the same P1 peptide as V5B2 [23]. These antibodies differ from V5B2 either in terms of their prion protein isoform specificities or other binding properties, as summarized in Table 1.

**Table 1:** The monoclonal antibodies used in the negative selection process.

**mAb**	**Form of mAb**	**Antigen used for mice immunization**	**Isotype**	**Specificity**	**Reference**
E12/2	Fab	recBoPrP	IgG1	PrP^C^, PrP^Sc^, α1-helix	[26]
C1/1	Fab	P1 (HuPrP: 214–226)	IgG1	PrP^Sc^, PrP^C^	[23]
K4H5	Fab	P1 (HuPrP: 214–226)	IgG1	PrP^Sc^	[23]
E9/5	IgG	P1 (HuPrP: 214–226)	IgG2a	PrP^Sc^	[23]

recBoPrP, recombinant bovine prion protein; HuPrP, human prion protein

**Figure 3:**
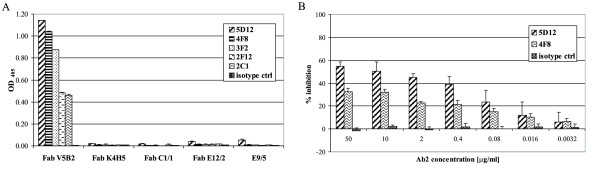
**Ab2 monoclonal antibody selection and the binding competition in ELISA**. (A) Positive and negative selection steps. Each column represents the immunoreactivity of individual Ab2 antibodies to the coated positive/negative selection antigens in the selection process. Each Ab2 antibody was used at 1 μg/ml. (B) Ab2 monoclonal antibody competition between the Ab2 antibody and the P1 peptide for the binding to V5B2. Each column represents the mean inhibition of at least three experiments ± standard deviation.

The nucleotide and amino-acid sequences of the variable domains of both the light and heavy chains of the anti-P1 antibodies were determined in our previous studies (manuscript in preparation). To compare not only their specificity, but also their structural determinants, the amino-acid identities and similarities between the variable regions of V5B2 and other anti-P1 monoclonal antibodies that were used for negative selection were examined. According to the identity and similarity matrices, the closest resemblance of V_L _and V_H _of V5B2 were seen with the C1/1 and K4H5 monoclonal antibodies. In particular, the V_L _of C1/1 was 85% identical and 91% similar to the V_L _of V5B2 and the V_H _of K4H5 was 90% identical and 93% similar to the V_H _of V5B2, with respect to the primary protein structures. The differences between these antibodies were predominantly restricted to the CDR regions only, as indicated by the frequencies of non-conserved substitutions (NCS) and insertions and deletions (INDEL) inside and outside these regions (Table 2). Still, all selected Ab2 antibodies exclusively recognize Fab V5B2 (Fig. 3A).

**Table 2:** Comparison of V5B2 and the antibodies used for the negative selection.

	**V5B2 V_H_**					**V5B2 V_L_**			
			
			**Frequency of NCS and INDEL**					**Frequency of NCS and INDEL**	
							
	**identity**	**similarity**	**inside CDR**	**outside CDR**		**identity**	**similarity**	**inside CDR**	**outside CDR**
**K4H5 V_H_**	0.902	0.927	0.16	0.01	**K4H5 V_L_**	0.534	0.698	0.61	0.09
**C1/1 V_H_**	0.685	0.774	0.28	0.06	**C1/1 V_L_**	0.847	0.910	0.16	0.01
**E9/5 V_H_**	0.457	0.630	0.31	0.09	**E9/5 V_L_**	0.590	0.718	0.28	0.10

^NCS, non-conserved substitutions; INDEL, insertions and deletions;^

Amino-acid identities and similarities between the variable regions of the heavy and light chains of the V5B2 monoclonal antibody, in comparison to the variable regions of the heavy and light chains of the other monoclonal antibodies used for negative selection. The amino-acid identities and similarities were calculated using the BLOSUM62 matrix.

In all, 44 hybridoma cell lines that were obtained in four cell fusions produced antibodies that reacted with Fab V5B2, although only five hybridoma cell lines corresponded to our strict selection criteria, all of which originated from mice immunized with the Fab fragment of V5B2 (see next section and Fig. 3A). According to the binding affinities and the capacity to inhibit the binding of V5B2 to the P1 peptide in ELISA, the two most potent Ab2 antibodies (5D12 and 4F8) are further described here. These selected Ab2 monoclonal antibodies had high affinities for Fab V5B2 in indirect ELISA. The *Kaff *determined were 8.04 ± 0.19 × 10^8 ^M^-1 ^and 1.2 ± 0.02 × 10^9 ^M^-1 ^for 5D12 and 4F8, respectively.

### Ab2 competition studies

Further characterization of the Ab2 monoclonal antibodies in terms of competitive studies showed concentration-dependent inhibition of V5B2 binding to the P1 peptide, with over 55% and over 35% inhibition in the presence of 50 μg/ml 5D12 and 4F8, respectively. The isotype control 6C5 monoclonal antibody did not cause any significant inhibition over a wide range of concentrations (Fig. 3B).

**Figure 4:**
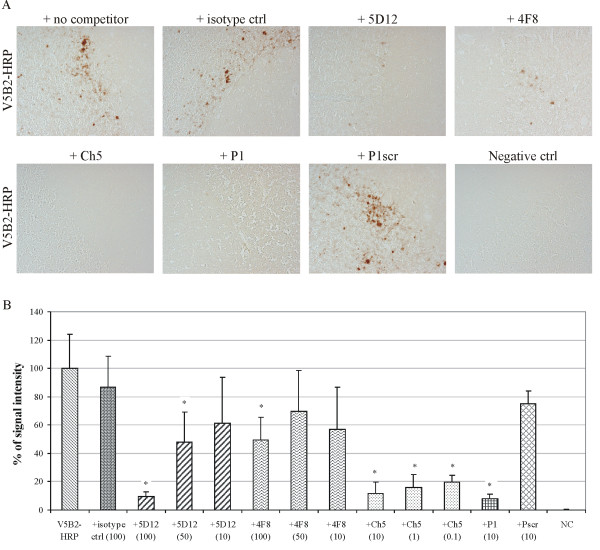
**Competitive immunohistochemical studies**. (A) Representative photomicrographs of immunohistochemically stained sCJD cerebellar cortex sections in the absence and presence of different competitor molecules. The brown precipitate in the Purkinje cells layer and internal granular cell layer indicates specific prion immunoreactivity to a variable degree in all of the images except the negative control. Only photomicrographs of sections stained with the use of following concentrations of competitor molecules are shown: 100 μg/ml for isotype control, 5D12 and 4F8, and 10 μg/ml for Ch5, P1 and Pscr. (B) Bar chart showing the effects of different competitor molecules at varying concentrations on the mean immunohistochemical signal intensity, expressed as percentages of the uncompeted V5B2-HRP immunoreactive signal intensity (positive control). Asterisks indicate statistically significant (p < 0.004) reductions in the mean signals. Competitor molecule concentrations are given in μg/ml in brackets.

Since the P1 peptide is only a mimic of the PrP^Sc^-specific epitope on the PrP^Sc ^molecule, and since the final target is the PrP^Sc ^molecule itself, we wanted to determine whether Ab2 antibodies can inhibit the binding of V5B2 to PrP^Sc ^directly in immunohistochemistry of brain slices of a sCJD affected individual. Fig 4 shows the results of immunohistochemical staining in the presence of different competitor molecules through representative photomicrographs of labelled tissue sections (Fig. 4A) and in a graphical form (Fig. 4B).

Preincubation of V5B2 with its original antigen, the P1 peptide (at 10 μg/ml), resulted in a 92% reduction in the signal relative to the positive control (p < 0.004), while the scrambled P1 peptide (Pscr) at the same concentration (10 μg/ml) caused a statistically non-significant decrease in comparison to the positive control. Preincubating V5B2 with the 5D12 and 4F8 Ab2 monoclonal antibodies at 100 μg/ml resulted in a 90% and 50% drop in the signal, respectively. Both of these effects were statistically significant (p < 0.004). A 50 μg/ml concentration of 5D12 still caused a significant, 52% drop in signal, whereas further dilution of 5D12 to 10 μg/ml had a lesser effect, reducing the signal by 39%, which did not reach statistical significance. The isotype negative control antibody at 100 μg/ml again produced a statistically non-significant effect compared to the positive control. All three concentrations of the purified Ch5 chicken polyclonal Ab (10, 1 and 0.1 μg/ml) caused large and highly significant (p < 0.004) signal decreases, of 88%, 84% and 81%, relative to the positive control.

### Sequencing of anti-idiotypic antibodies and 5D12-V5B2 molecular docking

The nucleotide and amino-acid sequences of V_H _and V_L _were determined [EMBL:AM850706, EMBL:AM850707 and EMBL:FM180446, EMBL:FM180447 for V_H _and V_L _of 5D12 and 4F8, respectively]. According to the results of the competitive studies, 5D12 was chosen as the most promising monoclonal Ab2 and its interaction with V5B2 was further characterized with molecular docking.

**Figure 5:**
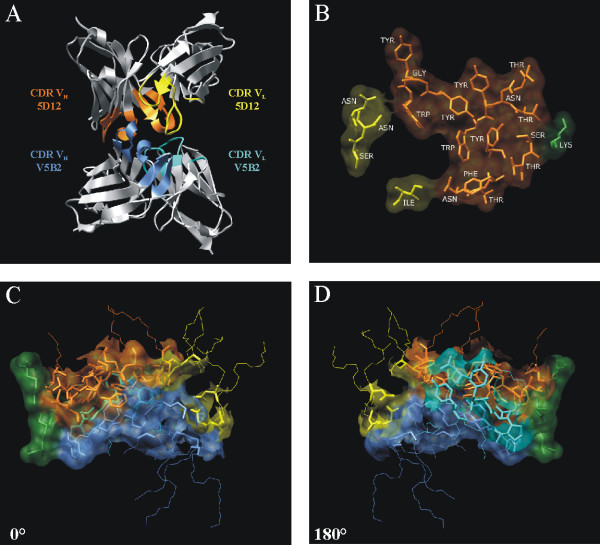
**The proposed model of the 5D12-V5B2 complex obtained with molecular docking**. (A) A ribbon diagram of the 5D12 and V5B2 variable regions illustrating the orientation of both binding partners with the CDR regions located at the molecular interface. (B) The amino-acid residues forming the putative 5D12 paratope. The residues from the V_L _and V_H _CDR regions are shown in yellow and orange, respectively. The only contact residue that lies outside of the CDR regions is coloured in green. (C) A semi-transparent depicition of the 5D12-V5B2 contact surface. The CDR loops from V_L _5D12, V_H _5D12, V_L _V5B2 and V_H _V5B2 are shown in yellow, orange, cyan and blue, respectively. Stick representation is used for all contact residues and wire representation of the backbone atoms is used for the remaining CDR residues. The contact residues that are not part of the CDR loops are coloured in green. The same representation, rotated for 180° around the vertical axis, is show in (D).

The Patchdock algorithm generated 3845 matches between the 5D12 and V5B2 models based on their surface complementarity. The 100 best solutions were further refined and scored using the Firedock algorithm. In the solution that shows the best surface complementarity, the molecular interface between 5D12 and V5B2 was formed by the CDR regions of both models (Fig. 5A). At the same time, this orientation had the 3^rd ^lowest global energy of all the matches scored using Firedock. The two solutions with the lower global energy only ranked 37^th ^and 34^th ^with respect to the complementarity and both proposed binding to the C-terminal face of the V5B2 model. As this surface is an artifact of the model, which contains only variable regions of both chains, and is not exposed in the entire mAb or Fab fragment, these two solutions were discarded as irrelevant.

The amino-acid residues that form the 5D12-V5B2 contact interface were identified as the ones, in which any atom of the residue is separated from any binding partner atom by a distance ≤ 4 Å. For both binding partners the majority of the contact residues were found to be located in the CDR loops: 19/20 and 18/20 contact residues in the case of 5D12 and of V5B2, respectively (Figs. 5C and 5D). With the exception of CDR-L2, all CDR loops from 5D12 contributed to the 5D12-V5B2 binding surface.

The comparison of the P1 peptide sequence (CITQYERESQAYY) and the 5D12 interacting surface residues revealed several similarities. The centre of the 5D12 interface is composed of aromatic residues, especially Tyr, which correspond to the C-terminal Tyr-Try motif of the P1 peptide. In addition the polar Ser and Thr residues, separated from the Tyr patch by an Asn residue resemble the Ser-Gln motif in the P1 peptide sequence (Fig. 5B).

## Discussion

Over the past decade, the anti-idiotypic vaccine approach has been successfully used for various aspects of vaccinology, such as autoimmune diseases, enteric intoxification, rejection of allografts, allergic diseases, foetal immunity regulation, and in particular, tumour immunotherapy [27]. This is the method of choice when the surrogate protective antigens are carbohydrate moieties or glycoproteins, when protective antigens are difficult to isolate and purify, when synthetic peptides cannot form the tertiary structure present in protective antigens, and when infectious agents with unpredictable effects are used [28-31]. Some of the properties listed also hold true for the pathological form of the prion protein. Induction of protective anti-prion immune response in wild type animals is extremely difficult, because of the host tolerance to endogenous PrP^C ^(for review see [21]). To date, no entirely successful attempt of active immunization in the field of TSE has been published, although some hypothetically protective target sites on the prion protein have been proposed [18,19]. However, to our knowledge, no method has been widely accepted. Anti-idiotypic vaccines would therefore represent a novel approach for prion disease prevention and treatment.

Results of passive vaccination and cell-culture studies indicate that only monoclonal antibodies that bind to the membrane PrP^C ^are efficient in blocking prion propagation and pathogenesis [32-35]; at the same time, it has been shown that monoclonal antibodies that recognized both PrP^C ^and PrP^Sc^, blocked PrP^Sc ^accumulation *in vitro *even more efficiently than monoclonal antibodies specific for PrP^C ^only [36]. However, the cross-linking of PrP^C ^*in vivo *caused extensive neuronal damage [37] and in our opinion this finding needs to be noted when potent PrP^C^-specific antibodies are to be induced or introduced for treatment or prophylaxis purposes. Therefore an approach to exclusively target PrP^Sc ^would avoid all of the problems regarding the binding of antibodies to PrP^C ^and the resultant cessation of normal PrP^C ^function.

To directly target the pathological isoform in the development of an anti-idiotypic vaccine, a PrP^Sc^-specific antibody is a prerequisite. However, only a few PrP^Sc^-specific antibodies have been reported [23,24,38-40], and due to their individual characteristics, not all of them are suitable for this purpose.

The V5B2 monoclonal antibody that was previously described by our group is a potent PrP^Sc^-specific antibody that reacts with the native PrP^Sc ^deposits in immunohistochemistry [24]. This thus represents an appropriate candidate to preserve the information of the native PrP^Sc^-specific epitope in its antibody-combining site. In the present study, the main ways of producing Ab2 antibodies against the PrP^Sc^-specific antibody have been studied in two experimental models: in xenogeneic and syngeneic systems.

Mammalian proteins are highly immunogenic in chickens. As expected for xenogeneic immunization, the antibody titre in the antiserum was high. Although the immune response was expected against the whole immunoglobulin molecule, anti-idiotypic response was surprisingly potent, with the ability to totally block the binding of V5B2 to the P1 peptide or PrP^Sc^. However, despite promising results after testing the immune sera and affinity purified Ab2 IgY antibodies, we were not able to isolate stable chicken hybridoma cell lines that retained the antibody production. The main reason appears to be that to date chicken hybridoma technology has been poorly studied in comparison to mouse hybridoma technology; the system therefore still needs to be optimized for use as a routine experimental procedure.

In our syngeneic studies, the antigen (Ab1 V5B2) was actually a self-protein, produced in a BALB/c mouse and injected into an inbred BALB/c mouse with virtually the same genetic background, and therefore self-tolerance to the whole constant part of the immunoglobulin was expected. Groups of BALB/c mice were immunized with 3 different forms of the same antigen. Whole IgG V5B2 was the original and the least chemically treated molecule used for immunization. It is a relatively large molecule, predominantly composed of the self constant region, with only a minor part being immunogenic in a syngeneic experimental system. Although the immune tolerance to the whole constant part of the immunoglobulin molecule was expected, we also wanted to assess whether the use of the smaller fragment Fab V5B2, lacking the constant Fc-region, potentiates the immune response to idiotopes. In addition to the whole V5B2 and Fab V5B2, the Fab V5B2, covalently coupled to the highly immunogenic carrier molecule KLH, was used for immunization, to maximize the specific helper T-cell response and to potentiate the immune response against idiotopes on V5B2. Nevertheless, a prominent immune response was achieved with all three antigens. Surprisingly, even though the immune response against the whole IgG V5B2 was relatively strong, the antisera capacity to inhibit the V5B2 binding to the P1 peptide was much lower in comparison to the inhibitory capacity of the antisera obtained by immunization with the smaller fragment, Fab V5B2. Because of the higher molecular weight of the whole IgG V5B2 in comparison to the Fab V5B2, less idiotopes of V5B2 are present per μg of antigen injected in case of the whole IgG. This could contribute to the lower immune response and lower inhibitory capacity of the obtained sera. It is also possible that the immune response was directed predominantly against the glycan moieties of the constant region in case of the whole IgG, as the glycosylation profile of monoclonal antibodies is affected by the cell culture conditions and can differ from the normal antibody glycosylation observed *in vivo *[41]. Based on the observed inhibitory capacity, only the mice immunized with Fab V5B2 and Fab V5B2-KLH were sacrificed for the cell fusion. The resulting hybridoma cell lines produced predominantly IgG Ab2 antibodies, in contrast to other Ab2 antibody studies, where the antibodies were mainly of the IgM class [4,5,42,43]. The obtained Ab2 antibodies bound Fab V5B2 with a high affinity, characteristic of affinity-matured IgGs.

The main objective in the present study was to select Ab2 antibodies that recognize private idiotopes. Therefore, the selection process included Fab fragments of monoclonal antibodies with the same specificities for the peptide antigen, but with different specificities for the prion protein isoforms. Indeed, the majority of the antibodies used in the negative selection process (Table 1) were directed against the P1 peptide derived from the C-terminal amino-acid sequence of the human prion protein, which can adopt different conformations in solution, and therefore, which can elicit antibodies of different specificities when injected into wild-type mice [23]. The comparison of the variable region amino-acid sequences of both the light and heavy chains, between antibodies that had been used in the negative selection process and our antigen, Fab V5B2, revealed considerable analogy. The V_H _and V_L _amino-acid sequences of these antibodies differ only slightly, and most importantly, the differences between these antibodies are predominantly restricted to the CDR regions (Table 2). Nevertheless, all of the Ab2 monoclonal antibodies selected still distinguished between the Fab fragments of these antibodies, which indicated a very precise selectivity of our Ab2 monoclonal antibodies for private idiotopes, most probably restricted to the CDR regions of V5B2. Subsequent *in silico *structural modelling of the 5D12-V5B2 complex also revealed the close interaction of both CDR regions (Fig. 5).

Based on the selection process described and the affinities of the Ab2 antibodies for Fab V5B2 as well as their inhibition potential, two Ab2 monoclonal antibodies, 5D12 and 4F8, were selected for further characterization. Since no functional test is available to determine the presence of Ab2 antibodies that bind to the antigen-combining site of a PrP^Sc^-specific antibody, at first an ELISA-based inhibition assay was designed to characterize the Ab2 antibody response. Ab2 antibodies in chicken and mouse polyclonal immune sera and Ab2 murine monoclonal antibodies (Figs. 1B, 1C, 2B and 3B) substantially inhibited the binding of Ab1 to the P1 peptide in a dose-dependent manner. This indicated that in agreement with idiotypic network theory, immunization with Fab V5B2 induced Ab2 antibodies, which bear the functional epitope for Ab1 that competes with the original antigen, the P1 peptide, and in turn have to be similar to the P1 peptide structure. The reasons for the stronger inhibition of the polyclonal immune sera in comparison to monoclonal antibodies can be accounted for by the higher antibody concentration, a wider variety of Ab2 antibody specificities, and consequently also a wider variety of target epitopes present in polyclonal preparations.

In addition to the ELISA-based inhibition assay, the competitive immunohistochemistry assay was set up to show that Ab2 antibodies inhibit not only binding of V5B2 to the P1 peptide but also to the original target molecule, PrP^Sc^. Indeed, we observed 90% and 50% inhibition of binding of V5B2 to PrP^Sc ^using high concentrations of 5D12 and 4F8, respectively, whereas the isotype control did not show any significant inhibition at the same concentrations. The inhibition of binding was concentration dependent, which indicates the specificity of the effects seen. Furthermore we saw 88% inhibition with the use of much lower concentrations of polyclonal Ch5 as competitor, again probably due to a wider variety of Ab2 antibody specificities with synergistic effects, present in the polyclonal preparation. The results of the competitive immunohistochemical studies, together with the results of the competitive ELISA, strongly indicate that the selected Ab2 antibodies bind into the V5B2 binding site, thereby hindering the binding of V5B2 to the P1 peptide, and most importantly to the original target, the PrP^Sc ^molecule. In addition, the structural model of the 5D12-V5B2 complex, obtained by molecular docking, also strongly supports the close interaction of both antibody-combining regions, since the majority of the interacting residues are located in the CDR regions (Figs. 5C, 5D).

Based on previous studies, Ab2 antibodies can resemble the amino-acid sequences of nominal antigens in their variable regions, especially when the nominal antigen is a peptide [44-46]. The P1 peptide motif could be preserved in a single CDR sequence or in a conformational stretch across different CDR loops of a single chain, as well as across different loops of both the heavy and light chains. The latter, i.e. the involvement of different CDR loops of both chains, is also evident from the proposed model of the 5D12-V5B2 complex. The comparison of the 5D12 binding interface to the P1 peptide reveals structural similarities to its C-terminal. This is especially important in the light of our recent unpublished data, which indicate that the C-terminal residues are crucial for the V5B2 binding to the P1 peptide. It is therefore possible that the 5D12 binding surface mimics the crucial part of the P1 peptide and the epitope, unique to the PrP^Sc ^molecule. Nevertheless, the computer modelling data must be interpreted cautiously; still, the obtained docking model is in agreement with the observed experimental data. The proposed model also achieved an outstanding ranking score using both the surface complementarity and the global energy criteria.

## Conclusion

With all of the data collected, we have shown that it is possible to induce a strong Ab2 antibody immune response against a PrP^Sc^-specific monoclonal antibody in both the xenogeneic and the syngeneic experimental systems. Among the three different antigens tested, the Fab fragment was the most successful form for the immunization of mice. In addition, we have demonstrated that it is possible to select Ab2 monoclonal antibodies specific for private idiotopes using a two-step selection process. In our case, the availability of related antibodies that predominantly differ in CDR regions only, enabled the critical selection step in the negative selection process. Ab2β, the internal image antibodies, are directed against the binding site of the eliciting antibody and are structural and/or functional mimics of the original antigen. According to the convincing results of the competition studies and the computer modelling data, 5D12 is a highly plausible candidate for being an internal image antibody, mimicking the PrP^Sc^-specific epitope. The results of the present study not only provide an example of the successful production of Ab2 monoclonal antibodies based on a well planned strategy for selection, but should also provide a new experimental approach that is applicable to the field of prion diseases.

## Methods

### Animals

Chickens from a divergently selected line (D+) for high body weight [47] were bred and maintained in the Department of Animal Science at the Biotechnical Faculty, University of Ljubljana. BALB/c mice were bred and maintained in the Animal Facility at the Blood Transfusion Centre of Slovenia. Both groups of animals were handled in accordance with the FELASA recommendations and guidelines.

### Cell lines

V5B2 (Ab1), a B-cell hybridoma cell line of BALB/c origin, was generated against the 13-residue synthetic P1 peptide (CITQYERESQAYY), which was derived from the C-terminal α-helix of the human prion protein (amino acids 214–226), coupled to keyhole limpet hemocyanin (KLH) [24]. The NS1 murine myeloma cell line, the V5B2 hybridoma cell line and all murine hybridoma cell lines prepared in this study were maintained in Dulbecco's modified Eagle's medium (DMEM; ICN Biomedical) supplemented with 13% bovine serum (HyClone), 2 mM L-glutamine (Sigma), 130 μg/μl streptomycin (Sigma) and 100 U/ml penicillin (Sigma) (subsequently referred to as DMEM+). The MuH1chicken myeloma cells and clones obtained by fusion of chicken cells were cultured in Iscove's Modified Dulbecco's medium (Sigma) with addition of foetal bovine serum (HyClone) and gentamycin (Sigma) at final concentrations of 10% and 0.1%, respectively.

### Antigens

We prepared three different antigens for the immunization: the whole V5B2 antibody, the Fab fragment of the V5B2 antibody, and the Fab fragment of the V5B2 antibody coupled to KLH. All supernatants from murine hybridoma cells were purified by gravity-flow affinity chromatography on Protein G Sepharose (Amersham) using 0.1 M glycine, pH 2.7, for elution. The Fab fragments were produced by cleaving with immobilized papain (Pierce) and purification on Protein A Sepharose CL-4B (Pharmacia Biotech). The identity and purity of the Fab fractions were determined by indirect ELISA and Western blotting. The Fab fragments of V5B2 were coupled to KLH via 4-maleimidobutyric acid N-hydroxysuccinimide ester (GMBS, Sigma), according to the procedure described by Yoshitake et al. [48].

### Chicken immunization and fusion protocol

Five chickens were immunized intramusculary every 3 weeks with the whole V5B2 monoclonal antibody as antigen. In each immunization, 400 μg of antigen was applied into three places in the breast muscle, in a total volume of 1 ml. For the first immunization, the antigen was mixed with Complete Freund's Adjuvant (1:1 v/v; Sigma), for the second immunization, with Incomplete Freund's Adjuvant (Sigma), and for all of the subsequent immunizations, with physiological saline. After the 4^th ^immunization, the titres of anti-V5B2 antibodies were estimated by ELISA and the two chickens with the highest antibody titres were selected. They received two more boosters of the Fab fragment of V5B2. Three days after the last immunization booster, these chickens were sacrificed, and their blood was collected for the preparation of the polyclonal anti-serum and their spleens were removed for monoclonal antibody production.

The chicken hybridoma cells were produced as described previously [49,50]. Briefly, spleen cells from the immunized chickens were fused with the MuH1 chicken myeloma cell line in a ratio of 1:3 using 50% polyethylene glycol. Hybridoma cells were selected while maintaining the cells in medium containing HAT (hypoxanthine, aminopterin, thymidine; Sigma) or HT (hypoxanthine, thymidine; Sigma), at a final concentration of 1%, and ouabain (Sigma), at a final concentration of 20 μM.

### Chicken polyclonal antibody affinity purification

Fab V5B2 was covalently immobilized to agarose beads (Pierce) according to the manufacturer protocol. Blood serum from the chicken with the most potent immune response, collected after immunization, was applied to the column with the immobilized Fab V5B2. After extensive washing the bound fraction was eluted using 0.1 M glycine, pH 2.7.

### Mouse immunization and fusion protocol

Female BALB/c mice (6–8 weeks old) were immunized with three different antigens: the whole V5B2 antibody, the Fab fragment of the V5B2 antibody, and the Fab fragment of the V5B2 antibody coupled to KLH. Each mouse was immunized subcutaneously with 0.1 mg antigen in Complete Freund's Adjuvant on day 0, and then intraperitoneally with 0.1 mg antigen in Incomplete Freund's Adjuvant on days 14, 28 and 42. On day 52, the mice were bled from the tail vein and the immune sera were collected. A final booster of 50 μg antigen in physiological saline was administered intravenously on day 57, although only into the mice with the highest antibody titres, which were the ones selected as spleen-cell donors for the cell fusion. The mice were sacrificed on day 61, and their spleens were removed. Splenocytes were isolated and fused with mouse NS1 myeloma cells with 50% polyethylene glycol for 3 min, according to standard procedures. Hybridoma cells were selected by maintaining the cells for two weeks in HAT DMEM+ and another week in HT DMEM+ selection medium (DMEM+ supplemented with HAT or HT, respectively). The presence of specific antibodies was determined in supernatants after 10 to 14 days by indirect ELISA. Selected hybridomas were cultured until stable cell lines were established, and then subcloned by limiting dilution, and frozen in liquid nitrogen.

### Murine anti-idiotypic monoclonal antibody selection

Microtitre ELISA plates (Nunc) were coated with 50 μl Fab fragment at 1 μg/ml in 50 mM carbonate/bicarbonate buffer, pH 9.6, and incubated overnight at 4°C. The next day, the plates were washed three times with washing buffer (sodium phosphate buffer, containing 150 mM NaCl, 0.05% Tween 20, pH 7.2–7.4) and blocked for 30 min at 37°C with blocking buffer (1% BSA in washing buffer). The plates were washed again and then incubated with the following primary antibodies: polyclonal immune sera, cell culture supernatant, or purified monoclonal antibodies, all diluted in blocking buffer, for 1.5 h at 37°C. After washing, the plates were incubated with a secondary goat anti-mouse IgG (γ-chain specific) conjugated to horseradish peroxidase (HRP, Sigma), diluted 1:3,000 in blocking buffer, and incubated again for 1.5 h at 37°C. After incubation with the 2,2'-azino-bis(3-ethylbenzothiazoline-6-sulfonic acid) (ABTS, Sigma) substrate in citrate-phosphate buffer, pH 4.5, for 20 min at 37°C, the reaction was measured spectrophotometrically at 405 nm with a microtitre plate reader.

The 5D12 and 4F8 clones were chosen for further experiments, as they consistently produced the highest titre antibodies, as determined by indirect ELISA. The 6C5 murine IgG1 monoclonal antibody, specific for antigen B of the human polysaccharide blood group system AB0, served as an isotype negative control monoclonal antibody throughout the study.

### Determination of affinity constants

The affinity constants (*Kaff*) of the Ab2 monoclonal antibodies were determined in a non-competitive immunoassay [51]. Briefly, an ELISA plate (Nunc) was coated overnight with Fab V5B2 at three different concentrations: 5 μg/ml, 2.5 μg/ml and 1.25 μg/ml. The Ab2 monoclonal antibodies were applied at three-fold serial dilutions, with concentrations ranging from 9 μg/ml down to 12 ng/ml. The incubations with the secondary antibody and the ABTS substrate were performed as described above.

### Competitive ELISA

#### Chicken immune sera

For the competition studies, ELISA plates (Nunc) were coated with 0.5 μg/ml P1 peptide (JPT) overnight. The next morning, the plates were washed and blocked as described above. Then 1 μg/ml V5B2 was separately mixed with different dilutions of chicken immune sera and incubated for 1 h at 37°C prior to application to the blocked ELISA plate, with a further incubation for 90 min at 37°C. This step was followed by washing and the addition of a secondary goat anti-mouse Fab-specific HRP conjugate (Sigma) diluted 1:5,000 in blocking buffer, with a further incubation for 1.5 h at 37°C. Detection was performed as above, and the percentage of specific inhibition was calculated relative to buffer controls and by taking the optical density of the well containing no competitor as 100%.

#### Mouse immune sera, mouse monoclonal antibodies and purified chicken polyclonal antibodies

As described above, ELISA plates were coated overnight at 4°C with 0.5 μg/ml or 0.25 μg/ml P1 for testing immune sera and purified antibodies, respectively. The next morning, the plates were washed and blocked with blocking buffer. V5B2 conjugated to HRP (V5B2-HRP), diluted 1:1,500, was separately mixed with different dilutions of mice immune sera, Ab2 monoclonal antibodies, purified chicken polyclonal antibodies (subsequently called Ch5) or an isotype control antibody, and incubated for 1 h at 37°C prior to application to the blocked ELISA plate and incubation for another 1 h at 37°C. After washing, the detection step was carried out as described above, and again, the percentage of specific inhibition was calculated relative to buffer controls and by taking the optical density of the well containing no competitor as 100%.

### Immunohistochemistry

#### Tissue samples

Adjacent sections of paraformaldehyde-fixed, paraffin-embedded human cerebellar tissue samples from a patient with confirmed sporadic CJD (sCJD) with synaptic prion deposition and primitive plaques (Val/Val on codon 129 of the prion protein gene) were used in the study, which were obtained from the archive of the Institute of Pathology, Faculty of Medicine, University of Ljubljana. The tissue samples were immersed in 96% formic acid for 1 h after fixing in paraformaldehyde. The 5-μm-thick sections were used immediately after deparaffination and the antigen retrieval procedure involving 30 min autoclaving at 121°C in distilled water and then a 5-min incubation in 96% formic acid, which is the standard recommended pretreatment for optimal PrP^Sc ^immunodetection in tissue sections [52].

#### Competitive immunohistochemistry

Direct immunohistochemical detection of prion deposits was performed with V5B2-HRP and using 3,3-diaminobenzidine as chromogen (Sigma). V5B2-HRP was diluted 1:100 in a vehicle solution containing 1% BSA (Sigma) and 0.04% Triton X-100 (Sigma) in phosphate buffer, pH 7.2. Competitors at stated concentrations were preincubated with V5B2-HRP in the same solution for 12 h at 4°C before applying the solution onto tissue sections, which were then incubated overnight in a moist chamber at room temperature. The sections were then rinsed thoroughly (10 × 10 min) and developed in chromogen for 5 min. Simultaneously processed sections incubated only in vehicle buffer solution and developed in chromogen served as a negative control.

### Image collection and analysis

Photomicrographs were taken using 20 × objective on a Nikon Eclipse E600 microscope equipped with a Nikon DXM 1200 digital camera, and connected to a personal computer station running NIS-D software. Every section was photographed at 5 locations, chosen to always reveal cerebellar cortical layers in balanced proportions.

Images of the brown immunoreactive product were smoothed with Gaussian kernel (1 pixel radius), inverted and colour-hue adjusted (the hue was rotated by 272°) to transform the brown immunoreactive product on a white background to a green signal on a black background. All of the image pre-processing was accomplished in Adobe Photoshop CS3 (Adobe, San Jose, CA, USA) using an automated action. All further analysis was conducted on the green signal intensity only. To establish a baseline from which signal was estimated, a threshold was chosen at which only 0.1% of the negative control image surface remained above threshold. All images were then thresholded (all pixels with intensity below the threshold were set to 0) and the mean signal value for each image was computed. To remove the effects of the remaining noise, the mean signal value of negative control images (n = 5) was subtracted from each image value. Finally, to express the signal intensities as percentages of the positive control intensity, the mean intensity values were divided by the mean positive control intensity (uncompeted immunohistochemistry with V5B2-HRP) and multiplied by 100. All of the image intensity analysis was carried out using Matlab (Mathworks, Natick, MA, USA).

### Statistical analysis of immunohistochemical data

The differences in effects of different competitor molecules were estimated using one-way ANOVA. To test for significant differences in intensities between test samples and the positive control, a one-tailed t-test for independent samples with unequal variances was used. Based on the number of tests, a Bonferroni corrected p value of 0.004 was chosen as the threshold for statistical significance.

### Polymerase chain reaction amplification and sequence analysis of heavy and light chains

Amplification and sequencing of monoclonal Ab2 antibody heavy and light chains were performed as described by Koren et al. [53]. All of the nucleotide sequence analyses and translations were carried out using the BioEdit programme [54] or the ExPASy proteomics server [55]. All of the immunoglobulin gene analyses were performed using IMGT, the international ImMunoGeneTics information system [56].

### Antibody modeling and molecular docking

The variable regions of V5B2 and 5D12 were modeled following the procedure described by Morea et al. [57]. For V5B2, relevant chains from Protein Data Bank (PDB) structures 1KB5 and 1NBV were used as the light and heavy chain variable regions (V_L _and V_H_) templates, showing 94.5% and 92.7% similarity to the corresponding V5B2 sequences, respectively. The H3 hypervariable loop was modeled separately, using H3 loop from the PDB structure 1VFA as the closest match of the same length and torso conformation, with 53.8% similarity. For 5D12, PDB structures 2PCP, 1FSK and 1DBA were used as the templates for V_L_, V_H _and H3 loop, with the similarities to the corresponding 5D12 sequences being 97.5%, 85.9% and 73.3%, respectively. All similarities were calculated using the BLOSUM62 matrix All structure manipulations were carried out in Swiss-PdbViewer [58] and UCSF Chimera [59].

The 5D12 and V5B2 variable region models were docked using Patchdock molecular docking algorithm based on shape complementarity principles [60], with the antibody-antigen complex type option enabled. The 5D12 model was specified as the antibody and the V5B2 model as the antigen. One hundred best ranking solutions were further refined and scored according to a binding energy function using the Firedock algorithm [61].

## Abbreviations

The abbreviations used are: ABTS: 2,2'-azino-bis(3-ethylbenzothiazoline-6-sulfonic acid); Ab2: anti-idiotypic; BSE: bovine spongiform encephalopathy; CDR: complementarity-determining region; DMEM: Dulbecco's modified Eagle's medium; DMEM+: DMEM supplemented with 13% bovine serum, 2 mM L-glutamine, 130 μg/μl streptomycin and 100 U/ml penicillin; GMBS: 4-maleimidobutyric acid N-hydroxysuccinimide ester; HAT: hypoxanthine, aminopterin, thymidine; HRP: horseradish peroxidase; HT: hypoxanthine, thymidine; HuPrP: human prion protein; INDEL: insertions and deletions; *Kaff*: affinity constant of an antibody; KLH: keyhole limpet hemocyanin; NC: negative control; NCS: non-conserved substitutions; PrP^C^: cellular isoform of prion protein; PrP^Sc^: pathological isoform of prion protein; Pscr: peptide with scrambled amino-acid sequence of the P1 peptide; recBoPrP: recombinant bovine prion protein; sCJD: sporadic Creutzfeldt-Jakob disease; TSE: transmissible spongiform encephalopathy; V5B2-HRP: peroxidase-conjugated monoclonal antibody V5B2; vCJD: variant Creutzfeldt-Jakob disease.

## Authors' contributions

ACV carried out the murine immunizations, antibody selection and characterization, participated in the design of the study and drafted the manuscript. MB participated in the design of competitive immunohistochemical studies and carried out the image collection, analysis and statistical analysis of immunohistochemical data. TV carried out the immunohistochemical study and participated in the design of the study. SK carried out the antibody modelling and molecular docking. MN carried out the chicken immunizations and cell fusion experiment. MP participated in evaluation of immunohistochemical results. VCS conceived of the study, and participated in its design and coordination and helped to draft the manuscript. All authors read and approved the final manuscript.
